# Chrononutrition is associated with melatonin and cortisol rhythm during pregnancy: Findings from MY-CARE cohort study

**DOI:** 10.3389/fnut.2022.1078086

**Published:** 2023-01-06

**Authors:** Ai Ni Teoh, Satvinder Kaur, Siti Raihanah Shafie, Nurul Husna Mohd Shukri, Normina Ahmad Bustami, Masaki Takahashi, Shigenobu Shibata

**Affiliations:** ^1^Faculty of Applied Sciences, UCSI University, Kuala Lumpur, Malaysia; ^2^Department of Nutrition, Faculty of Medicine and Health Sciences, Universiti Putra Malaysia, Serdang, Selangor, Malaysia; ^3^Faculty of Medicine and Health Sciences, School of Healthy Aging, Medical Aesthetics and Regenerative Medicine, UCSI University, Kuala Lumpur, Malaysia; ^4^Institute for Liberal Arts, Tokyo Institute of Technology, Tokyo, Japan; ^5^Department of Electrical Engineering and Biosciences, School of Advanced Engineering and Sciences, Waseda University, Tokyo, Japan

**Keywords:** circadian rhythm, melatonin, cortisol, chrononutrition, dietary pattern, breakfast skipping, late-night eating, gestation

## Abstract

Chrononutrition has been suggested to have an entrainment effect on circadian rhythm which is crucial for metabolic health. Investigating how chrononutrition affects maternal circadian rhythm can shed light on its role during pregnancy. This study aims to determine chrononutrition characteristics of healthy primigravida during pregnancy and its association with melatonin and cortisol rhythm across gestation. A total of 70 healthy primigravidas were recruited from ten randomly selected government maternal and child clinics in Kuala Lumpur, Malaysia. During the second and third trimesters, chrononutrition characteristics including meal timing, frequency, eating window, breakfast skipping, and late-night eating were determined using a 3-day food record. Pregnant women provided salivary samples at five time-points over a 24 h period for melatonin and cortisol assay. Consistently across the second and third trimesters, both melatonin and cortisol showed a rhythmic change over the day. Melatonin levels displayed an increment toward the night whilst cortisol levels declined over the day. Majority observed a shorter eating window (≤12 h) during the second and third trimesters (66 and 55%, respectively). Results showed 23 and 28% skipped breakfast whereas 45 and 37% ate within 2 h pre-bedtime. During the third trimester, a longer eating window was associated with lower melatonin mean (β = –0.40, *p* = 0.006), peak (β = –0.42, *p* = 0.006), and AUC_G_ (β = –0.44, *p* = 0.003). During both trimesters, a lower awakening cortisol level was observed in pregnant women who skipped breakfast (β = –0.33, *p* = 0.029; β = –0.29, *p* = 0.044). Only during the second trimester, breakfast-skipping was significantly associated with a greater cortisol amplitude (β = 0.43, *p* = 0.003). Findings suggest that certain chrononutrition components, particularly eating window and breakfast skipping have a significant influence on maternal melatonin and cortisol rhythm. Dietary intervention targeting these characteristics may be useful in maintaining maternal circadian rhythm.

## 1. Introduction

There is a growing interest in chrononutrition, which studies the timing of food in relation to circadian rhythm alignment. Feeding cues have been found to predominantly entrain peripheral clocks in organs such as kidney and liver, which then interact with the master clock to keep the body’s circadian rhythm synchronized (1). Food intake is naturally circadian, where energy stores are replenished through food intake during the active phase while fasting takes place during the sleep phase. Mistimed feeding in individuals at hours outside the normal sleep/wake cycle under a regular light/dark cycle can induce a disruption in metabolism (2, 3), which has been shown to be closely related to circadian rhythm (4). For instance, spontaneous food intake at the end of the resting phase (late afternoon) results in the most detrimental effects on energy homeostasis in obese rodents, whereas for humans, this critical period is at the early night or the beginning of the resting phase (5). Irregular feeding patterns such as delayed meal intake, breakfast skipping and late-night eating have been associated with circadian rhythm disruption, ranging from delayed glucose rhythms (6), phase delay in core body temperature (7), delayed peripheral clock genes (8) and blunted cortisol secretion (9).

During pregnancy, the food intake of women in terms of quality and quantity is a well-recognized determinant of maternal and infant outcomes, such as birth weight, gestation length and gestational weight gain (10). However, less is known about the relationship between maternal chrononutrition characteristics with pregnancy and infant outcomes. A recent review by Loy et al. (11) summarized that night eating during pregnancy was linked to metabolic implications, including the risk of gestational diabetes mellitus (GDM), undesired gestational weight gain pattern, postpartum weight retention and preterm birth. A more recent randomized controlled trial using a chrononutritional and sleep hygiene intervention showed improvement in maternal glycemic control among pregnant women with gestational diabetes mellitus (GDM) (12). This evidence linking chrononutrition with metabolic health and adverse birth outcomes suggests that the timing aspect of food intake during gestation may have a wider influence on the maternal physiological system, which is circadian in nature (13). Given the growing body of evidence demonstrating the association between disrupted circadian rhythm and poor pregnancy outcomes (14–16), unfavorable chrononutrition characteristics can be a potential modifiable approach to improve maternal and infant outcomes by intervening the temporal aspect of food intake.

To date, most studies involving the general adult population have shown the relationship between various chrononutrition aspects and adverse health outcomes, such as diabetes, cardiovascular diseases and obesity (17, 18). In the pregnant population, the impact of varying chrononutrition on maternal health has yet to be fully elucidated. Identifying the chrononutrition characteristics associated with maternal circadian rhythm during pregnancy can provide practical insights into the modifiable aspects of maternal nutrition through food intake, which can be targeted for maternal health promotion. Thus, this study aimed (1) to determine maternal circadian rhythm in terms of melatonin and cortisol levels during the second and third trimesters; (2) to identify maternal chrononutrition characteristics during the second and third trimesters; (3) to determine the association of maternal melatonin and cortisol levels with chrononutrition characteristics during the second and third trimesters. It is hypothesized that unfavorable chrononutrition characteristics including longer eating window, breakfast skipping and late-night eating are linked to differences in maternal melatonin and cortisol levels during the second and third trimesters of pregnancy.

## 2. Materials and methods

### 2.1. Study design and data collection

The results described in this paper were derived from a larger MY-CARE observational cohort study conducted to determine the effect of maternal circadian rhythm during gestation on birth and infant outcomes (19). Between June 2019 to October 2021, healthy primigravidas with singleton pregnancy, aged between 19 and 39 years, and in their first 20 weeks of gestation took part in the study. Subject recruitment was carried out at ten randomly selected government maternal and child clinics (*Klinik Kesihatan Ibu dan Anak*) in Kuala Lumpur, Malaysia using purposive sampling. Pregnant women with the following conditions were excluded from the study: pre-existing health conditions such as diabetes mellitus, hypertension, and anemia, pregnancy-related complications, physical disabilities, consuming medicine or supplement containing melatonin or corticosteroids, using sleep medicine or recreational drugs, smoking, take part in shift work, or undertook transmeridian flight in the past 3 months upon recruitment.

The sample size needed for the study was calculated using G*POWER software, version 3.1.9.4^1^. Using a linear multiple regression model, effect size (f^2^) of 0.338 (20), type I error rate (α) of 0.05 and power (1-β) of 90%, the minimum sample size required was 49. The required sample size was increased by 20% to 60 considering attrition and non-compliance. The final number of pregnant women after excluding dropout and non-compliance was 70. The flow of subject recruitment and participation was depicted in Supplementary material.

The socio-demographic characteristics of the pregnant women, such as age, race, educational level and household income level were collected using a face-to-face questionnaire at the clinic upon recruitment. Pre-pregnancy weight and height were obtained from the antenatal booklet to compute pre-pregnancy body mass index (BMI). Data collection was conducted only during the second and third trimesters with exclusion of the first trimester as pregnant women are normally in their late first trimester or early second trimester (week 9–12) at their first antenatal check-up.

### 2.2. Ethics approval and consent to participate

Pregnant women provided signed written informed consent before data collection commenced. The study was carried out in accordance with The Malaysian Code of Responsible Conduct in Research and the Helsinki Declaration. Ethical approval for the study was obtained from the Medical Research and Ethics Committee (KKM/NIHSEC/P19-125) on 29^th^ April 2019.

### 2.3. Chrononutrition characteristics

The timing of food intake, types of food, beverage and supplement and the amount consumed were recorded using a 3-day food record. Pregnant women were instructed to record their food intake on three non-consecutive days (two weekdays and one weekend day) in order to capture differing eating patterns on weekdays and weekends. To reduce reporting bias among the pregnant women, a one-on-one briefing on the usage of the 3-day food record was conducted and visual aid of portion size using household measurements was provided along with the food record. To minimize recall bias, pregnant women were advised to record their food intake immediately after each eating event. A review of the food entries was conducted upon returning the 3-day food record. The ratio between reported total energy intake (EI) and basal metabolic rate (BMR) was calculated to identify underreporters. Underreporting was defined as having an EI:BMR ratio <1.2 (21) whereas an EI:BMR ratio of >2.4 was considered overreporting (22). BMR was calculated using the equation for Malaysian adults with the additional energy requirements during pregnancy (second trimester: 280 kcal/day, third trimester: 470 kcal/day) (23). In this study, 8.6% (*n* = 6) and 14.3% (*n* = 10) of pregnant women underreported their energy intake in the second and third trimesters, respectively. Hence, 64 and 60 pregnant women were included in the final analysis.

Chrononutrition characteristics that were assessed in this study include meal timing, meal frequency, eating window, largest meal, breakfast-skipping and late-night eating. Based on the food entries on the 3-day food record, mean meal timing for the main meals (breakfast, lunch, and dinner) and snacks was computed. Meal frequency is defined as the average number of meals and snacks consumed in a day. Eating window refers to the duration of time in minutes between the first eating event and the last eating event of the day (24). The largest meal of the day was identified based on the amount of calories consumed. In this study, breakfast is defined as the first meal of the day consumed no later than 10:00 h (25). Breakfast consisting of tea or water only was excluded (26). Pregnant women were categorized as breakfast skippers (ate breakfast ≤2 out of the 3 days of food record) or breakfast eaters (ate breakfast on all 3 days). Lunch, dinner and snacks were self-defined by the pregnant women. Pregnant women who ate within 2 h before sleep were categorized as late-night eaters (late-night eating ≥1/3 days) (24, 27).

### 2.4. Salivary melatonin and cortisol levels

Salivary samples were collected for a day during the second and third trimester, respectively. Pregnant women collected at least 3 ml of salivary samples using passive drool method to determine their melatonin and cortisol levels. Pregnant women were instructed to collect their first salivary sample of the day upon awakening, followed by 9:00 h and at every 6 h interval (15:00, 21:00, and 3:00 h) within a 24 h day. These timings were selected in reference to previous studies and to capture the changes in melatonin and cortisol secretion, particularly in the morning post-awakening, mid-day, before going to sleep and mid-sleep (28, 29). They were briefed on the collection protocol at the clinics prior to home collection.

Pregnant women were requested to carry out their salivary sampling on a work-free day when they can freely choose their sleep and wake timings and follow their usual routine without the constraint of work commitment. Additionally, they were advised to avoid the intake of chocolate, banana, alcohol, caffeine or drinks containing artificial colorants on the sampling day. The consumption of prescribed over-the-counter medications within 12 h prior to sampling was required to be reported. Pregnant women were asked to avoid consuming a major meal or brushing teeth with toothpaste within 30 min prior to sampling. After collection, pregnant women stored their samples in their home freezer until collection by the researcher.

The samples were analyzed using direct salivary melatonin enzyme-linked immunosorbent assay (ELISA) kits from IBL International (Hamburg, Germany) and direct saliva cortisol ELISA kit from LDN Labor Diagnostika Nord GmbH & Co. KG (Nordhorn, Germany) according to the manufacturer’s instructions. Each sample was measured in duplicate to generate an average concentration value. Samples with concentrations that exceeded the standard range of the ELISA kit were diluted 2-fold and re-analyzed. The intra-assay and inter-assay coefficient of variation were 2.4 and 13.0% for melatonin measurements and 3.4 and 13.9% for cortisol measurements.

### 2.5. Chronotype

Chronotype of the pregnant women was assessed using the Morningness-Eveningness Questionnaire (MEQ). The MEQ was administered once upon recruitment and was not assessed repeatedly considering that chronotype is a stable trait with minor advances over years (30). The chronotypes of pregnant women were categorized by the following cut-offs: definite to moderate morning (score = 59–86), intermediate (score = 42–58), and definite to moderate evening (score = 16–41) (31).

### 2.6. Statistical analysis

All statistical analyses were conducted using SPSS software version 20 (SPSS Inc., Chicago, IL, USA). Continuous data with a normal distribution were reported as mean and standard deviation (SD) whereas median and interquartile range (IQR) were reported for data with a skewed distribution. Categorical variables were reported in percentage. Outliers and skewness of data distribution were determined using the Shapiro Wilk test. Outliers were excluded from analyses. A *p-*value of <0.05 was considered statistically significant. To check for the differences in melatonin and cortisol measurements between the second and third trimesters, paired sample *t*-test (for parametric variables) and Wilcoxon’s sign rank test (for non-parametric variables) were conducted.

Based on the four melatonin levels across the day (9:00, 15:00, 21:00, and 3:00 h), mean, maximal level, amplitude, area under the curve with respect to ground (AUC_G_), and area under the curve with respect to increase (AUC_I_) were computed. The maximal level is determined based on the highest melatonin level among the four collection timings. Amplitude is calculated as the ratio of the maximal melatonin level to the lowest melatonin level. It is commonly examined as a marker that indicates the robustness of melatonin secretion across the day. Both AUC_G_ and AUC_I_ were tabulated using the trapezoid method (32). The AUC_G_ represents the total melatonin production across the day, whereas AUC_I_ indicates the increment of melatonin secretion over the 24 h day. A more positive value of melatonin AUC_I_ indicates a steeper increment in melatonin level across the day. Melatonin level at z-awakening was not analysed in this study as the aim of collecting salivary sampling at awakening was to determine awakening cortisol level, which is a commonly examined feature of cortisol rhythm but not for melatonin rhythm.

On the other hand, mean, awakening level, amplitude, diurnal slope, morning slope, evening slope, and AUC_G_ were calculated based on the five salivary samples analysed for cortisol levels. Amplitude and AUC_G_ of cortisol were calculated based on the same method for melatonin. Diurnal cortisol slope was calculated by dividing the difference in the cortisol levels at 9:00 and 21:00 h by the difference in hours, representing the diurnal cortisol decline. To further examine the variation in the rate of cortisol decline in the morning and from afternoon to the evening, morning slope (difference in the cortisol levels at 9:00 and 15:00 h over difference in hours) and evening slope (difference in the cortisol levels at 15:00 and 21:00 h over difference in hours) were calculated. A more negative slope represents a steeper rate or slope of cortisol decline over the period, whereas a more positive slope represents a flatter decline in cortisol level.

In this study, potential covariates that may be associated with salivary melatonin and cortisol levels include study design-related factors (gestation week at sampling), maternal socio-demographic characteristics [maternal age, race, household income level, educational level, and pre-pregnancy body mass index (BMI)], situational factors (wake and sleep time on sampling day and sleep duration), and fetal sex. Preliminary analyses were conducted using univariate tests to examine the univariate associations between the abovementioned variables and salivary melatonin and cortisol parameters. Variables that showed significant association were included as controls in the subsequent analyses.

Hierarchical linear regression analyses were performed to examine the association of maternal melatonin and cortisol variables with chrononutrition characteristics while adjusting for covariates. The potential covariates identified from the preliminary analyses were entered in the first step, whereas chrononutrition variables were entered in the second step of the hierarchical regression model. Entering the independent variables and covariates by the enter method allows the examination of the incremental predictive effect of chrononutrition variables on outcome variables. Collinearity between variables entered in the model was tested with variance inflation factors (VIFs), where VIF greater than five indicates collinearity.

## 3. Results

### 3.1. Subject characteristics

Mean age of pregnant women in this study was 28.7 years old. The majority was Malay (*n* = 39, 55.7%) and attained tertiary education (*n* = 59, 84.3%). Around 47.1% (*n* = 33) came from households with middle income. In terms of pre-pregnancy BMI, 10.0% (*n* = 7) were underweight, whereas 22.8% (*n* = 16) were overweight or obese before pregnancy. The majority are of intermediate chronotype (71.4%), with only 4.3% are evening chronotype. The subject characteristics were summarized in Table 1.

**TABLE 1:** Characteristics of the study sample (*n* = 70).

	Mean	SD	Range	*n*	%
Age (years)	28.7	3.7	20–37	—	–
Gestational week (weeks)	–	–	–	–	–
T2	20.0	3.7	13–27	–	–
T3	32.9	2.1	28–37	–	–
Pre-pregnancy BMI (kg/m^2^)	23.3	4.9	15.8–42.3	–	–
Underweight	–	–	–	7	10.0
Normal	–	–	–	47	67.1
Overweight	–	–	–	12	17.1
Obese	–	–	–	4	5.7
Ethnicity	–	–	–	–	–
Malay	–	–	–	39	55.7
Chinese	–	–	–	26	37.1
Indian	–	–	–	2	2.9
Others	–	–	–	3	4.3
Educational level	–	–	–		
Secondary	–	–	–	11	15.7
Tertiary	–	–	–	59	84.3
Household income (RM)*	–	–	–	–	–
Low (<2300)	–	–	–	7	10.0
Middle (2300–5599)	–	–	–	33	47.1
High (≥5600)	–	–	–	30	42.9
MEQ score	53.9	6.8	–	–	–
Morningness	–	–	–	17	24.3
Intermediate	–	–	–	50	71.4
Eveningness	–	–	–	3	4.3

BMI, body mass index; RM, ringgit Malaysia; T2, second trimester; T3, third trimester.

*Based on the definition of 10^th^ Malaysia Plan.

### 3.2. Circadian variation in salivary melatonin level

**FIGURE 1:**
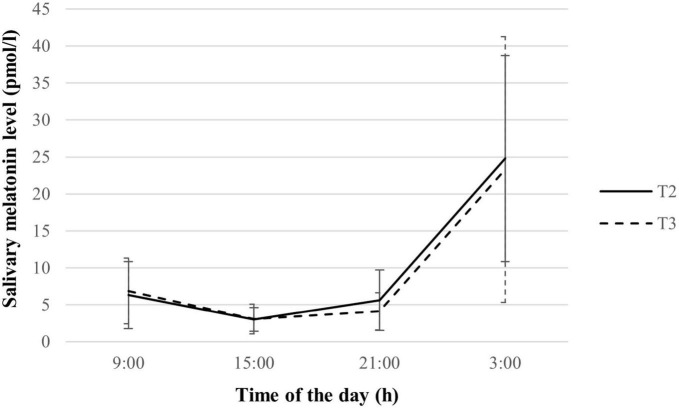
Salivary melatonin levels of pregnant women during the second and third trimesters (*n* = 70). Values are expressed in median ± median absolute deviation. T2, second trimester; T3, third trimester.

Melatonin levels and variables were listed in Supplementary material. A distinct change in melatonin levels across the days was observed (See Figure 1). Melatonin secretion showed a significant increment from 21:00 to 3:00 h, with relatively low levels at 9:00 and 15:00 h in both the second and third trimesters. No significant difference in melatonin levels across all collection timings (all *p* > 0.05). The melatonin rhythm variables including mean, maximal level, AUC_G_, and AUC_I_ were maintained across trimesters (all *p* > 0.05). Melatonin amplitude was significantly lower in the third trimester as compared to the second trimester (*p* = 0.020), indicating an attenuated melatonin response over the day.

### 3.3. Circadian variation in salivary cortisol levels

**FIGURE 2:**
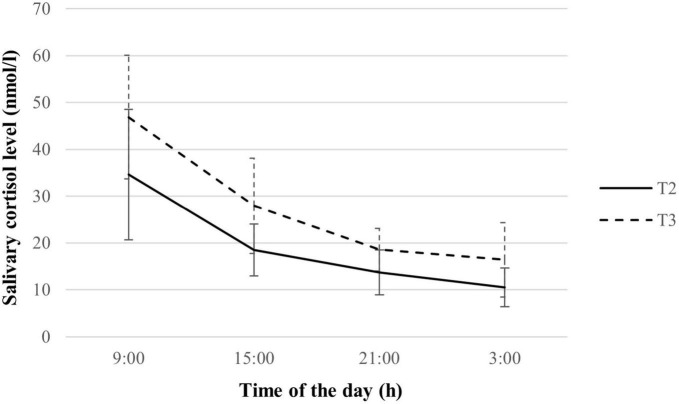
Salivary cortisol rhythm of study subsample during the second and third trimesters (*n* = 70). Values are expressed in median ± median absolute deviation. T2, second trimester; T3, third trimester.

Pregnant women showed a distinct decline in cortisol levels over the day during the second and third trimesters of pregnancy, as depicted in Supplementary material. An overall increase in cortisol levels in the third trimester compared to the second trimester was observed (See Figure 2), with significant differences in cortisol levels across all collection timings (all *p* < 0.05). Mean and AUC_G_ of cortisol were also significantly higher in the third trimester (both *p* < 0.001), indicating a greater melatonin output. However, there was no significant variation in the amplitude and patterns of cortisol decline, namely diurnal slope, morning slope, evening slope, and AUC_I_ as pregnancy advanced.

### 3.4. Chrononutrition characteristics

Chrononutrition characteristics of pregnant women were listed in Table 2. Meal timing, frequency and eating window did not significantly vary by trimester. The majority of the pregnant women followed an eating window of 12 h or below both in the second (65.6%, *n* = 42) and third (55.0%, *n* = 33) trimesters. A higher proportion of pregnant women in this study reported having lunch or dinner as their largest meal of the day. In the third trimester, the prevalence of breakfast skipping was significantly higher (*p* = 0.005), whereas the prevalence of late-night eaters was significantly lower than in the second trimester (*p* = 0.002).

**TABLE 2:** Chrononutrition characteristics of the pregnant women during second (*n* = 64) and third trimester (*n* = 60).

Variable	T2 (*n* = 64)	T3 (*n* = 60)	*P*-value
***n* (%)/Mean** ± **SD**			
Meal timing			
Breakfast	8:41 ± 0:47	8:32 ± 0:43	0.510
Morning snack	10:37 ± 0:54	10:20 ± 0:39	0.427
Lunch	13:18 ± 0:55	13:11 ± 0:58	0.320
Afternoon snack	16:22 ± 1:07	16:25 ± 1:02	0.301
Dinner	19:54 ± 0:55	19:57 ± 0:52	0.876
Supper	22:41 ± 1:18	22:25 ± 1:07	0.827
Meal frequency	4.45 ± 0.79	4.28 ± 0.78	0.079
Eating window	11.95 ± 1.43	11.91 ± 1.63	0.708
≤12 h	42 (65.6)	33 (55.0)	–
12–14 h	18 (28.1)	20 (33.3)	–
>14 h	4 (6.3)	7 (11.7)	–
Largest meal^a^	–	–	0.104
Breakfast	9 (14.1)	7 (11.7)	–
Morning snack	1 (1.6)	3 (5.0)	–
Lunch	25 (39.1)	26 (43.4)	–
Afternoon snack	1 (1.6)	–	–
Dinner	28 (43.8)	24 (40.0)	–
Supper	–	–	–
Breakfast-skipping	–	–	0.005**
Breakfast eater	49 (76.6)	43 (71.7)	–
Breakfast skipper	15 (23.4)	17 (28.3)	–
Late night eating	–	–	0.002**
Non-late-night eater	35 (54.7)	38 (63.3)	–
Late-night eater	29 (45.3)	22 (36.7)	–

Independent *t*-test was carried out for continuous variables while McNemar’s test was used for categorical variables. Paired sample *t*-test was conducted to determined differences between second and third trimesters based on sample’s data. ^a^Fisher’s exact test was performed as expected count less than five was more than 20%. SD, standard deviation; T2, second trimester; T3, third trimester. **p < 0.01.

### 3.5. Association between maternal melatonin levels and chrononutrition characteristics

As shown in Table 3, significant associations were found between longer eating window and lower mean (β = –0.40, *p* = 0.006), peak (β = –0.42, *p* = 0.006), and AUC_G_ (β = –0.44, *p* = 0.003) of melatonin rhythm in the third trimester. Each of them explains an additional 14.4, 15.2, and 17.3% of the variance. No significant association was observed in the second trimester. Results for the non-significant models were listed in Supplementary material.

**TABLE 3:** Hierarchical linear regression models predicting maternal melatonin parameters from chrononutrition characteristics (T2: *n* = 64; T3: *n* = 60).

	Melatonin during T3					
	Mean		Maximal level		AUC_G_	
	β (95% CI)	*P*-value	β (95% CI)	*P*-value	β (95% CI)	*P*-value
**Step 1:**						
Maternal age	0.07 (–0.55, 0.92)	0.613	–0.04 (–2.09, 1.53)	0.756	0.12 (–0.97, 2.43)	0.388
Pre-pregnancy BMI	0.24 (–1.36, 9.30)	0.140	0.19 (–5.32, 20.50)	0.242	0.23 (–3.58, 20.73)	0.162
Gestational week	–0.01 (–1.39, 1.30)	0.950	–0.12 (–4.59, 1.86)	0.397	0.10 (–1.95, 4.13)	0.473
Fetal sex	0.07 (–4.23, 7.07)	0.615	0.03 (–12.24, 14.99)	0.839	–0.09 (–16.93, 8.89)	0.533
Sleep time	–0.30 (–5.43, 0.19)	0.067	–0.29 (–12.84, 0.81)	0.082	–0.27 (–11.89, 0.99)	0.096
ΔR^2^ for step 1	0.126	0.306	0.136	0.302	0.087	0.541
**Step 2:**						
Eating window	–0.40 (–4.07, 0.72)	0.006**	–0.42 (–9.76, 1.72)	0.006**	–0.44 (–9.61, –2.09)	0.003**
ΔR^2^ for step 2	0.144	0.006**	0.152	0.006**	0.173	0.003**
F_model_	2.592	0.032*	2.625	0.031*	2.474	0.039*

AUC_G_, area under the curve with respect to ground; β, standard coefficients; BMI, body mass index; T3, third trimester. **p < 0.01; *p < 0.05.

### 3.6. Association between maternal cortisol levels and chrononutrition characteristics

In both the second and third trimesters, a lower awakening cortisol level was observed in pregnant women who skipped breakfast (T2: β = –0.33, *p* = 0.029; T3: β = –0.29, *p* = 0.044), explaining an additional variance of 8.0 and 14.7% of the variance (See Table 4). Only during the second trimester, breakfast-skipping was significantly associated with a greater cortisol amplitude (β = 0.43, *p* = 0.003). It explains an additional 13.5% of the variance. Results for the non-significant models were listed in Supplementary material.

**TABLE 4:** Hierarchical linear regression models predicting maternal cortisol parameters from chrononutrition characteristics (T2: *n* = 64; T3: *n* = 60).

	Cortisol during T2				Cortisol during T3	
	Awakening		Amplitude		Awakening	
	β (95% CI)	*P*-value	β (95% CI)	*P*-value	β (95% CI)	*P*-value
**Step 1:**						
Maternal age	–0.11 (–1.95, 0.92)	0.474	–0.01 (–0.15, 0.16)	0.967	0.09 (–1.28, 2.44)	0.534
Pre-pregnancy BMI	0.18 (–0.48, 2.31)	0.195	–0.22 (–0.30, 0.02)	0.085	0.01 (–1.73, 1.82)	0.960
Household income level	0.32 (0.65, 16.68)	0.035*	0.18 (–0.33, 1.51)	0.201	0.14 (–5.44, 15.26)	0.345
Gestational age	0.18 (–0.48, 2.35)	0.192	0.23 (–0.02, 0.29)	0.090	–0.14 (–4.84, 1.64)	0.324
Fetal sex	–0.19 (–16.91, 2.66)	0.150	0.05 (–0.86, 1.32)	0.679	–0.18 (–21.53, 4.13)	0.179
Wake time	0.02 (–3.61, 4.26)	0.868	–0.29 (–0.96, –0.07)	0.025*	0.41 (2.60, 13.26)	0.004**
ΔR^2^ for step 1	0.170	0.147	0.156	0.158	0.088	0.110
**Step 2:**						
Breakfast-skipping						
No	1.00		1.00		1.00	
Yes	–0.33 (–25.37, –1.47)	0.029*	0.43 (0.78, 3.55)	0.003**	–0.29 (–30.77, –0.41)	0.044*
ΔR^2^ for step 2	0.080	0.029*	0.135	0.003**	0.147	0.044*
F_model_	2.285	0.043	3.045	0.009**	2.306	0.042*

β, standard coefficients; BMI, body mass index; T2, second trimester; T3, third trimester. **p < 0.01; *p < 0.05.

## 4. Discussion

The results from this study provided an overall understanding of maternal chrononutrition characteristics and circadian rhythm in terms of melatonin and cortisol levels during the second and third trimesters of pregnancy. Overall, the mean salivary melatonin and cortisol level in this sample was approximately eight times lower than one study that measured salivary melatonin [Shimada et al. (33)] over two time points among healthy pregnant women. As compared to a previous study that reported a significant rise in serum melatonin levels as pregnancy advanced, with levels in the third trimester being around 1.4 to 3-fold higher, the overall mean melatonin levels in the present study did not differ significantly between the second and the third trimester (34–37). Increment of cortisol levels in both trimesters were approximately 2–3 times higher than the observations in previous studies (38–41).

Findings of the present study suggested that certain chrononutrition characteristics, particularly breakfast skipping and eating window can influence maternal melatonin and cortisol rhythm during pregnancy after controlling for covariates. Pregnant women who skipped breakfast were associated with a lower awakening cortisol level both during the second and third trimesters. Trimester-specific associations were also detected. Only during the second trimester, breakfast skippers showed a significantly higher cortisol amplitude. Additionally, a longer eating window during the third trimester was associated with an overall lower melatonin output in comparison to several studies [Nakamura et al. (35)], as measured by mean, peak and AUC_G_. Lowered melatonin levels during pregnancy have been shown to have negative effects by increasing risks of pre-eclampsia and intrauterine growth retardation (35).

The timing of the major meals (breakfast, lunch, and dinner), snacks, and supper overlaps with the typical meal patterns reported by other studies (42–44), which indicates that the intake of major meals are typically within a certain time frame across geography. A higher proportion of the pregnant women in this study had their lunch or dinner as the largest meal of the day, which is consistent with previous studies that reported a higher proportion of energy consumed at lunch and dinner, i.e., during the later times of the day (45, 46). The prevalence of breakfast-skipping in this study was at the upper end as compared to the previously reported prevalence among pregnant women (6–31%) (47–49). Pregnant women tend to be vulnerable to meal skipping due to factors such as morning sickness, sensitivity to smell, and reduced appetite (50–52). Furthermore, in this study, pregnant women who skipped breakfast had a significantly later wake and sleep time on both workdays and free days as well as a lower mean MEQ score or greater eveningness than breakfast eaters, implying that this eating behavior can be related to their chronotype preference. Taken together, the patterns of delayed food intake and breakfast skipping may also be related to the characteristics of the study sample, which largely comprised of working pregnant women living in the urban city. In an urban environment, work and social commitments, eating out culture and around-the-clock dining are factors that likely promote higher energy intake toward the later times of the day and breakfast-skipping (53, 54). This phenomenon may not be unique to the current study sample but is prevalent in urban population.

On the other hand, around 33–46% of the pregnant women in this study are late-night eaters or ate within 2 h before sleep. Late-night eating and breakfast-skipping are typically seen in individuals with an evening tendency due to a collective delay in behaviors, including food intake (55). Further analysis showed no significant correlation between breakfast eating or chronotype with night eating in this study. This may be because the majority of pregnant women reporting night eating in this study were due to the consumption of maternal milk at night before sleep instead of eating a major meal. In addition, with the majority of pregnant women consuming their largest meal either in the afternoon or at night, it is possible that they are more likely to consume food closer to bedtime due to an overall delayed food intake pattern.

In this study, a longer eating window, which implies a shorter fasting cycle relative to the feeding cycle, was associated with an overall reduced output of melatonin rhythm, namely mean, peak and AUC_G_. The association between a longer eating window and lower melatonin secretion may be a result of a circadian mismatch between the active-feeding/resting-fasting cycle and the circadian regulation of metabolic physiological processes. The study of eating windows is based on time-restricted eating (TRE) which involves limiting daily food intake to 8–12 h to prolong a fasting period (56). Aligning the feeding/fasting cycle to hormonal regulation across the 24 h day means restricting energy intake to the active phase of the day whereas stored energy is used during the resting phase (57). This result can be explained by the role of melatonin in regulating the daily distribution of metabolic processes and hormones to synchronize the active/feeding phase to the high insulin sensitivity phase and the resting/fasting phase to the insulin-resistant phase of the day (57, 58). Previous studies examining the effect of fasting on melatonin found reduced nocturnal melatonin levels (59, 60) and phase advance of melatonin rhythm in response to fasting (61). As the results from the aforementioned studies were participants who were subjected to 48–72 h of fasting, they may not be directly comparable with the results in this study. However, taken together, these findings support the hypothesis that altering the length of the feeding/fasting cycle can have an impact on circadian melatonin secretion.

The association between eating window and melatonin rhythm in this study appeared to be trimester specific. As the median and patterns of eating window did not significantly vary across trimester, the trimester-specific association may be related to the physiological changes that occur as pregnancy advances. An example of physiological changes as demonstrated in this study is the elevation of cortisol level in the third trimester. Melatonin and cortisol are physiologically linked, and both play various key roles in circadian system and energy homeostasis along with other hormones, such as insulin, leptin, and ghrelin. Although melatonin levels remain relatively stable across trimester, the larger, collective physiological changes may potentially confound the trimester-specific association. Nonetheless, the mechanistic pathway underlying this trimester-specific association requires further study. Based on the results, it remains unclear what is the desirable range of eating window to maintain melatonin secretion. Majority of the pregnant women in this study had an eating window of 8–12 h. Further study is recommended to examine the different range of eating window and its influence on circadian melatonin secretion.

Additionally, it was found that pregnant women who skipped breakfast had a significantly higher cortisol amplitude during the second trimester and a lower awakening cortisol level during both the second and third trimesters. These associations between breakfast-skipping and cortisol level or amplitude have been reported previously. A study conducted by Witbracht et al. (62) reported that both non-stimulated and meal-stimulated cortisol levels over the day in women who regularly skip breakfast were elevated, particularly at midday. Furthermore, a smaller morning to evening cortisol amplitude or a blunter diurnal cortisol response was observed. These results were contrary to the findings in the present study where an increased amplitude and reduced awakening level were found. It is plausible that the lower cortisol awakening level and higher cortisol amplitude observed among breakfast skippers is due to delayed cortisol rhythm, where the increment in diurnal cortisol level occurs later post-awakening as compared to non-breakfast skippers. Breakfast-skipping is linked to evening chronotype and later sleep/wake timing (63, 64), which are associated with a delayed circadian phase (65–67). This explanation is supported by the observation that breakfast-skippers in this study had a significantly later wake and sleep time as well as a greater eveningness during the second and third trimesters.

Contrary to previous studies that demonstrated an association between night eating syndrome (NES) or late eating (lunch at 16:30) and differences in circadian melatonin and cortisol measurements such as lower amplitude and delayed circadian phase (9, 68), this study did not find such association. Delayed melatonin and cortisol rhythm along with blunted melatonin and cortisol secretion are prominent characteristics of circadian misalignment (69). The inconsistency in findings between the previous studies and the current study may be due to the difference in the definition of late-night eating, which in this study is defined as eating within 2 h before sleep. Examining the pattern and caloric intake of late-night eating may help to provide more comprehensive insights into its influence on circadian rhythm.

The current study findings should be interpreted in light of its limitations. Although the use of a 3-day food record comprising two weekdays and one weekend day is representative of the individual’s usual food intake, it may not be sufficient to capture daily variability in eating behaviors. Furthermore, the 3-day food record was self-reported by the participants. Hence there was a possibility of reporting bias. Salivary sampling was conducted at specific time-points in a 24 h day, which may not be adequate to capture individual variability in circadian melatonin and cortisol secretion, particularly with respect to the circadian peak and nadir. Furthermore, as both the salivary samplings and 3-day food record were not conducted on the same day, the associations found in this study may be confounded by factors contributing to day-to-day variability that were not studied in this research. The findings may not be generalized to other populations as the study was conducted among the urban pregnant women population. However, the study has several important strengths. Measuring both melatonin and cortisol levels as markers of circadian rhythm provides a more comprehensive understanding of the patterns of maternal circadian rhythm during pregnancy. It also allows the examination of the differential association of chrononutrition with melatonin and cortisol. Potential covariates of melatonin and cortisol secretion were also taken into account in the regression models. This study collected data during both the second and third trimesters which allow the detection of trimester-specific associations among the same pregnant women. Besides, this study focused on healthy primigravida, which would reduce the confounding effect of pregnancy-related complications on dietary intake and circadian rhythm.

## 5. Conclusion

Findings of this study suggested that certain chrononutrition characteristics, specifically breakfast skipping and longer eating window can contribute to significant lowered melatonin secretion and awakening cortisol levels among pregnant women. This indicates the potential influence of meal timing and pattern in altering maternal circadian rhythm, which is linked with disparities in pregnancy outcomes. Dietary intervention may need to consider incorporating chrononutrition characteristics on top of food quantity and quality to promote optimal pregnancy outcomes through maintaining circadian rhythm.

## Data availability statement

The raw data supporting the conclusions of this article will be made available by the authors, without undue reservation.

## Ethics statement

The studies involving human participants were reviewed and approved by Medical Research and Ethics Committee (KKM/NIHSEC/P19-125). The patients/participants provided their written informed consent to participate in this study.

## Author contributions

ANT was responsible for data collection, data analyses, and writing the manuscript. SK, NHMS, and SRS were responsible for the conception of the study, provided input to data analysis, and reviewed the manuscript. NAB, MT, and SS guided in lab analysis results and reviewed the manuscript with substantial contribution to the interpretation of the results. All authors approved the final version of the manuscript.

## Footnotes

1
http://www.gpower.hhu.de
