# Supplementary material for: Chrononutrition is associated with melatonin and cortisol rhythm during pregnancy: Findings from MY-CARE cohort study
Source: Front Nutr. 2023 Jan 6;9:1078086. doi: 10.3389/fnut.2022.1078086 (PMC9852999; doi:10.3389/fnut.2022.1078086)
Supplement: Supplementary file 1 [file Data_Sheet_1.docx]

| Groups | Seizure types or AED treatment | Chao1 index | |  | Faith's PD index | |
| --- | --- | --- | --- | --- | --- | --- |
|  |  | Tukey test P-value | ANOVA P-value |  | Tukey test P-value | ANOVA P-value |
| Seizure types | | | | | | |
| EP | Partial-Generalized | 0.76 | 0.49 |  | 0.81 | 0.39 |
|  | West_syndrome-Generalized | 0.70 |  |  | 0.53 |  |
|  | West_syndrome-Partial | 0.46 |  |  | 0.36 |  |
| ED | Partial-Generalized | 1.00 | 0.96 |  | 0.84 | 0.78 |
|  | West_syndrome-Generalized | 0.96 |  |  | 0.86 |  |
|  | West_syndrome-Partial | 0.96 |  |  | 0.98 |  |
| PT | Partial-Generalized | 0.82 | 0.09 |  | 0.94 | 0.56 |
|  | West_syndrome-Generalized | 0.13 |  |  | 0.64 |  |
|  | West_syndrome-Partial | 0.08 |  |  | 0.54 |  |
| Concomitant AEDs | | | | | | |
| EP | TPM-OXC | 0.91 | 0.44 |  | 0.89 | 0.35 |
|  | LTC-OXC | 0.89 |  |  | 0.79 |  |
|  | ACTH-OXC | 0.96 |  |  | 0.93 |  |
|  | Concomitant-OXC | 0.83 |  |  | 0.82 |  |
|  | LTC-TPM | 0.63 |  |  | 0.49 |  |
|  | ACTH-TPM | 0.68 |  |  | 0.59 |  |
|  | Concomitant-TPM | 0.46 |  |  | 0.41 |  |
|  | ACTH-LTC | 1.00 |  |  | 0.99 |  |
|  | Concomitant-LTC | 1.00 |  |  | 0.99 |  |
|  | Concomitant-ACTH | 1.00 |  |  | 1.00 |  |
| ED | TPM-OXC | 0.78 | 0.11 |  | 0.88 | 0.45 |
|  | VPA-OXC | 0.92 |  |  | 0.87 |  |
|  | ACTH-OXC | 0.44 |  |  | 1.00 |  |
|  | Concomitant-OXC | 0.49 |  |  | 0.5 |  |
|  | VPA-TPM | 1.00 |  |  | 1.00 |  |
|  | ACTH-TPM | 0.15 |  |  | 0.86 |  |
|  | Concomitant-TPM | 0.97 |  |  | 0.92 |  |
|  | ACTH-VPA | 0.3 |  |  | 0.83 |  |
|  | Concomitant-VPA | 1.00 |  |  | 1.00 |  |
|  | Concomitant-ACTH | 0.09 |  |  | 0.6 |  |
| PT | TPM-OXC | 1.00 | 0.14 |  | 1.00 | 0.29 |
|  | VPA-OXC | 0.57 |  |  | 0.99 |  |
|  | LTC-OXC | 0.74 |  |  | 1.00 |  |
|  | Concomitant-OXC | 0.49 |  |  | 0.31 |  |
|  | VPA-TPM | 0.62 |  |  | 0.98 |  |
|  | LTC-TPM | 0.77 |  |  | 1.00 |  |
|  | Concomitant-TPM | 0.74 |  |  | 0.64 |  |
|  | LTC-VPA | 1.00 |  |  | 1.00 |  |
|  | Concomitant-VPA | 0.18 |  |  | 0.5 |  |
|  | Concomitant-LTC | 0.27 |  |  | 0.6 |  |

**Table S1** Comparison of α-diversity indices between different seizure types or AED treatment. The α-diversity indexes were calculated with ANOVA and Tukey's multiple comparisons test.

**Table S2** NST and βNTI of the groups included in the study. All values were an average from all replicates ± standard deviations. Values with the same letters within a column did not significantly differ based on the Tukey's multiple comparisons test.

|  | NST | | | | βNTI | | | |
| --- | --- | --- | --- | --- | --- | --- | --- | --- |
|  | CK | EP | ED | PT | CK | EP | ED | PT |
| Minimum | -0.1767 | -0.4274 | -0.2737 | -0.07787 | -1.102 | -1.336 | -2.351 | -1.299 |
| Maximum | 0.6568 | 0.9053 | 0.9907 | 0.9983 | 6.311 | 6.544 | 5.339 | 7.379 |
| Range | 0.8335 | 1.333 | 1.264 | 1.076 | 7.413 | 7.879 | 7.69 | 8.678 |
| Mean±SD | 0.17±0.26**b** | 0.17±0.27**b** | 0.46±0.28**a** | 0.46±0.30**a** | 1.63±2.53**a** | 1.02±1.80**a** | 0.03±1.33**b** | 0.70±1.80**ab** |
| SEM | 0.056 | 0.03026 | 0.03209 | 0.03382 | 0.5526 | 0.2032 | 0.1502 | 0.2033 |

**Table S3** The relative abundance at phylum genus level of different groups. All values were an average from all replicates ± standard deviations. Values with the same letters within a column did not significantly differ based on the Tukey's multiple comparisons test.

|  | CK | EP | ED | PT |
| --- | --- | --- | --- | --- |
| Phylum (n, Mean±SD) |  |  |  |  |
| Acidobacteria | 0.035±0.021 | 0.006±0.015 | 0.008±0.01 | 0.015±0.022 |
| **Actinobacteria** | 36.719±26.103**ab** | 46.958±34.043**a** | 18.806±25.056**b** | 54.11±23.593**a** |
| Bacteroidetes | 0.001±0.003 | 1.05±1.827 | 5.078±11.506 | 5.268±12.922 |
| Cyanobacteria | 0.04±0.068 | 0.005±0.009 | 0.007±0.011 | 0.005±0.008 |
| **Firmicutes** | 36.519±33.277**ab** | 39.831±34.875**a** | 23.632±13.719**b** | 30.491±20.386**ab** |
| **Proteobacteria** | 26.683±18.66**b** | 5.151±4.651**c** | 52.427±19.936**a** | 8.94±6.967**bc** |
| Verrucomicrobia | 0.002±0.003 | 6.995±16.167 | 0.039±0.056 | 1.169±2.953 |
| genus (n, Mean±SD) |  |  |  |  |
| *Akkermansia* | 0.002±0.003 | 6.995±16.167 | 0.039±0.056 | 1.169±2.953 |
| *Bacteroides* | 0.001±0.002 | 0.925±1.558 | 4.943±11.347 | 5.208±12.889 |
| ***Bifidobacterium*** | 34.397±26.494**b** | 41.979±33.141**ab** | 17.732±24.982**c** | 50.676±23.068**a** |
| *Clostridium_sensu_stricto* | 4.144±5.61 | 1.273±3.477 | 9.469±12.572 | 0.048±0.065 |
| *Collinsella* | 0.001±0.001 | 4.626±11.119 | 0.026±0.086 | 0.989±2.433 |
| *Enterobacter* | 0.773±1.247 | 0.104±0.249 | 5.449±12.023 | 0.281±0.507 |
| *Enterococcus* | 1.632±2.948 | 5.738±20.092 | 1.318±2.955 | 0.15±0.264 |
| ***Escherichia/Shigella*** | 20.258±19.04**b** | 3.365±3.683**c** | 42.762±19.259**a** | 3.052±3.101**c** |
| *Faecalibacterium* | 0.026±0.046 | 9.84±20.415 | 0.026±0.04 | 0.779±1.88 |
| *Klebsiella* | 2.489±4.645 | 0.696±1.188 | 1.717±3.367 | 3.498±7.331 |
| *Lachnospiracea_incertae_sedis* | 10.614±27.995 | 10.498±24.359 | 3.336±5.969 | 1.58±2.794 |
| *Lactobacillus* | 3.052±4.84 | 0.261±0.582 | 0.169±0.305 | 8.686±16.665 |
| Others | 7.478±6.467 | 8.399±10.739 | 5.729±5.017 | 8.072±9.084 |
| *Streptococcus* | 6.533±3.137 | 3.367±6 | 3.794±4.879 | 14.219±13.428 |
| *Veillonella* | 8.608±18.32 | 1.946±2.767 | 3.482±4.44 | 1.588±2.945 |

**Table S4** Properties of the microbiota association networks in different groups

| **Properties of the networks** | **CK** | **EP** | **ED** | **PT** |
| --- | --- | --- | --- | --- |
| Nodes^a^ | 100 | 67 | 41 | 53 |
| Edges^b^ | 405 | 62 | 40 | 78 |
| Average degree distribution^c^ | 8.1 | 1.851 | 1.951 | 2.943 |
| Average clustering coefficient^d^ | 0.477 | 0.37 | 0.609 | 0.391 |
| Average path length^e^ | 3.333 | 3.587 | 2.339 | 3.804 |
| Modularity^f^ | 0.547 | 0.858 | 0.775 | 0.662 |
| Modules | 6 | 18 | 12 | 8 |

^a^ Number of AVSs with the Spearman correlation |r| > 0.8 and P-value < 0.05. ^b^ Number of significant (P-value < 0.05) correlations between nodes. ^c^ The larger the average distribution, the more complex the network distribution. ^d^ How nodes were embedded in their neighborhood, and the degree to which nodes tend to cluster together. ^e^ The capability of the nodes to form highly connected communities. ^f^ Modularity > 0.4 suggested that the network has a modular structure.

**Table S5** Genera and relative abundance of microorganisms at all nodes in the association network

| **Group** | **Genus** | **Relative abundance (%)** |
| --- | --- | --- |
| **CK** |  |  |
|  | *Bifidobacterium* (ASV_3, ASV_10, ASV_17, ASV_36) | 24.78 |
|  | *Escherichia/Shigella* (ASV_1) | 15.91 |
|  | *Lachnospiracea* incertae sedis (ASV_6) | 8.00 |
|  | *Lactobacillus* (ASV_19) | 5.44 |
|  | *Klebsiella* (ASV_49) | 2.03 |
|  | *Clostridium sensu stricto* (ASV_16) | 1.92 |
|  | *Veillonella* (ASV_35) | 1.73 |
| **EP** |  |  |
|  | *Lachnospiracea* incertae sedis (ASV_6) | 9.08 |
|  | *Akkermansia* (ASV_12) | 6.57 |
|  | *Enterococcus* (ASV_14) | 5.65 |
|  | *Escherichia/Shigella* (ASV_1) | 2.81 |
|  | *Streptococcus* (ASV_8, ASV_45) | 2.46 |
|  | *Bacteroides* (ASV_39, ASV_40) | 1.82 |
|  | *Clostridium* XlVa (ASV_26) | 1.56 |
|  | *Megasphaera* (ASV_25) | 1.38 |
| **ED** |  |  |
|  | *Bifidobacterium* (ASV_3, ASV_5, ASV_17) | 5 |
|  | *Enterobacter* (ASV_137) | 2.79 |
|  | *Kluyvera* (ASV_32, ASV_43) | 1.87 |
|  | *Clostridium* sensu stricto (ASV_54, ASV_187) | 1.7 |
|  | *Veillonella* (ASV_34) | 0.79 |
|  | *Streptococcus* (ASV_69) | 0.67 |
| **PT** |  |  |
|  | *Bifidobacterium* (ASV_2, ASV_4, ASV_5, ASV_17, ASV_24) | 16.6 |
|  | *Streptococcus* (ASV_8) | 9.38 |
|  | *Rothia* (ASV_27) | 2.02 |
|  | *Veillonella* (ASV_23) | 1.82 |
|  | *Klebsiella* (ASV_49) | 1.31 |
|  | *Lachnospiracea* incertae sedis (ASV_6) | 1.28 |
