# Supplementary material for: Chrononutrition is associated with melatonin and cortisol rhythm during pregnancy: Findings from MY-CARE cohort study
Source: Front Nutr. 2023 Jan 6;9:1078086. doi: 10.3389/fnut.2022.1078086 (PMC9852999; doi:10.3389/fnut.2022.1078086)
Supplement: Supplementary file 2 [file Data_Sheet_2.docx]

Supplementary Material

# Supplementary Figures

**
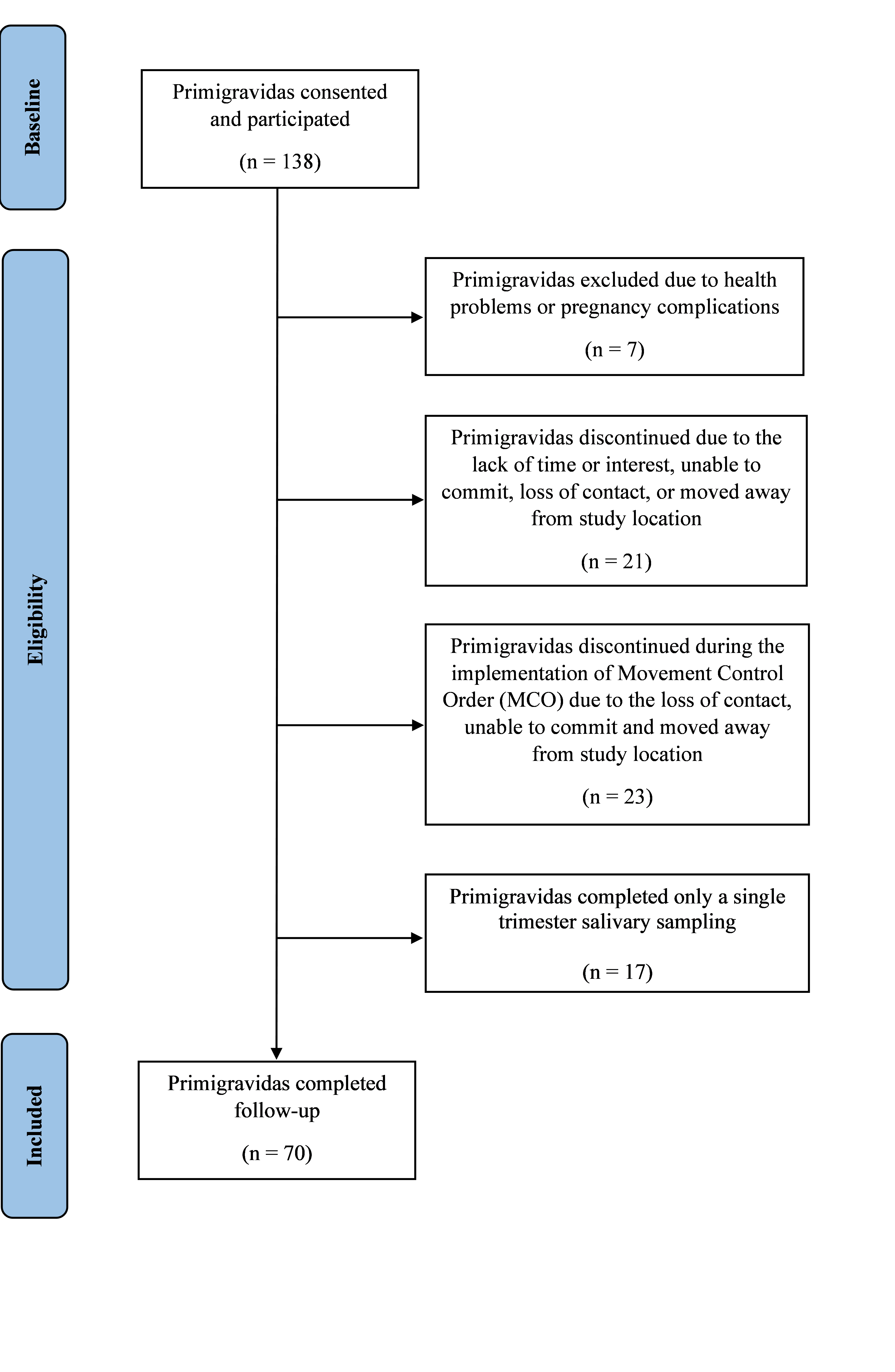
**

**Supplementary Figure 1.** Flowchart of recruitment and participation of pregnant women in this study.

# Supplementary Tables

**Supplementary Table 1.** Salivary melatonin levels of the study subsamples (n=70).

|  | **T2** | **T3** | ***p*-value** |
| --- | --- | --- | --- |
|  | **Mean ± SD/ Median (IQR)** | |  |
| Average sleep time (hh:mm)^b^ | 23:40 ± 1:07 | 24:03 ± 1:09 | 0.011* |
| Melatonin at 09:00 (pmol/l)^a^ | 6.34 (3.42, 6.34) | 7.35 (3.41, 12.94) | 0.379 |
| Melatonin at 15:00 (pmol/l)^a^ | 3.09 (1.40, 4.86) | 3.07 (1.19, 5.77) | 0.215 |
| Melatonin at 21:00 (pmol/l)^a^ | 5.62 (2.33, 11.47) | 4.20 (1.60, 8.73) | 0.420 |
| Melatonin at 03:00 (pmol/l)^a^ | 26.68 (10.90, 44.72) | 20.27 (6.06, 43.83) | 0.695 |
| Mean (pmol/l)^b^ | 12.74 (8.85, 19.95) | 11.89 (4.27, 19.84) | 0.161 |
| Amplitude^b^ | 31.19 (12.5, 46.45) | 12.62 (5.38, 27.57) | 0.020* |
| Maximal level (pmol/l/)^b^ | 31.30 (17.58, 48.84) | 22.24 (9.73, 44.24) | 0.719 |
| AUC_G_ (pmol/l)^b^ | 33.90 (23.04, 53.32) | 28.32 (10.51, 45.38) | 0.087 |
| AUC_I_ (pmol/l/h)^b^ | 12.94 (-5.59, 32.47) | 6.43 (-1.23, 28.99) | 0.586 |

Abbreviations: AUC_G_ = area under the curve with respect to ground; AUC_I_ = area under the curve with respect to increase; IQR = interquartile range; SD = standard deviation; T2 = second trimester; T3 = third trimester. **p*<0.05.

**Supplementary Table 2.** Salivary cortisol measurements of the study subsamples (n=70).

|  | **T2** | **T3** | ***p*-value** |
| --- | --- | --- | --- |
|  | **Mean ± SD/ Median (IQR)** | |  |
| Cortisol at awakening (nmol/l)^a^ | 35.77 (20.59, 52.04) | 43.81 (25.26, 56.45) | 0.011* |
| Cortisol at 09:00 (nmol/l)^a^ | 34.60 (21.64, 49.55) | 47.29 (33.71, 61.25) | 0.008** |
| Cortisol at 15:00 (nmol/l)^a^ | 18.44 (13.03, 24.47) | 27.68 (19.86, 39.15) | <0.001*** |
| Cortisol at 21:00 (nmol/l)^a^ | 13.92 (9.49, 19.53) | 18.96 (14.12, 24.44) | <0.001*** |
| Cortisol at 03:00 (nmol/l)^a^ | 12.04 (6.47, 17.20) | 15.82 (9.32, 27.22) | 0.009** |
| Average awakening time (hh:mm)^b^ | 7:46 ± 1:13 | 7:52 ± 1:11 | 0.375 |
| Mean (nmol/l)^b^ | 21.08 ± 7.36 | 28.60 ± 10.32 | <0.001*** |
| Amplitude^b^ | 5.02 ± 2.13 | 4.92 ± 2.43 | 0.972 |
| Diurnal cortisol slope (nmol/l/h)^b^ | -1.61 ± 1.41 | -1.86 ± 2.11 | 0.160 |
| Morning slope (nmol/l/h)^b^ | -2.47 (-4.88, -1.04) | -1.56 (-2.73, 0.46) | 0.674 |
| Evening slope (nmol/l/h)^b^ | -0.80 ± 1.41 | -1.26 ± 2.02 | 0.320 |
| AUC_G_ (nmol/l/h)^b^ | 57.87 ± 19.90 | 89.35 ± 34.76 | <0.001*** |

Abbreviations: AUC_G_ = area under the curve with respect to ground; AUC_I_ = area under the curve with respect to increase; SD = standard deviation; T2 = second trimester; T3 = third trimester.

Note: *p*-values are for the difference between T2 and T3 cortisol measurements calculated using the two-sample Wilcoxon signed rank test^a^ (for non-normally distributed data) and paired sample t-test^b^. **p*<0.05; ***p*<0.01; ***p<0.001

**Supplementary Table 3.** Hierarchical linear regression predicting melatonin rhythm from chrononutrition characteristics during pregnancy.

| **Melatonin rhythm during T2 (n = 70)** | | | | | | | | | | |
| --- | --- | --- | --- | --- | --- | --- | --- | --- | --- | --- |
| Variable | **Mean melatonin** | | **Amplitude** | | **Maximal** | | **AUC_G_** | | **AUC_I_** | |
|  | β (95% CI) | *p*-value | β (95% CI) | *p*-value | β (95% CI) | *p*-value | β (95% CI) | *p*-value | β (95% CI) | *p*-value |
| **Meal frequency** | 0.11 (-1.43, 3.43) | 0.412 | 0.13 (-4.13, 11.04) | 0.363 | 0.09 (-4.93, 9.74) | 0.513 | 0.05 (-5.69, 8.48) | 0.695 | 0.02 (-9.17, 10.65) | 0.881 |
| ΔR^2^ | 0.010 | 0.412 | 0.014 | 0.363 | 0.007 | 0.513 | 0.002 | 0.695 | 0.000 | 0.881 |
| **Eating window** | -0.05 (-1.61, 1.15) | 0.739 | -0.14 (-6.82, 2.31) | 0.325 | -0.21 (-7.04, 0.93) | 0.130 | -0.03 (-4.59, 3.53) | 0.795 | -0.17 (-9.67, 2.38) | 0.230 |
| ΔR^2^ | 0.002 | 0.739 | 0.017 | 0.325 | 0.036 | 0.130 | 0.001 | 0.795 | 0.024 | 0.230 |
| **Breakfast-skipping** |  |  |  |  |  |  |  |  |  |  |
| No | 1.00 |  | 1.00 |  | 1.00 |  | 1.00 |  | 1.00 |  |
| Yes | 0.02 (-5.01, 5.56) | 0.918 | 0.10 (-11.59, 21.20) | 0.558 | 0.10 (-10.92, 20.82) | 0.533 | -0.04 (-17.23, 13.17) | 0.790 | -0.04 (-25.41, 19.74) | 0.802 |
| ΔR^2^ | 0.000 | 0.918 | 0.006 | 0.558 | 0.006 | 0.533 | 0.001 | 0.790 | 0.001 | 0.802 |
| **Late-night eating** |  |  |  |  |  |  |  |  |  |  |
| No | 1.00 |  | 1.00 |  | 1.00 |  | 1.00 |  | 1.00 |  |
| Yes | -0.12 (-5.85, 2.09) | 0.346 | -0.20 (-20.47, 2.61) | 0.126 | -0.20 (-20.82, 2.44) | 0.119 | 0.03 (-10.10, 13.06) | 0.798 | 0.16 (-6.68, 25.79) | 0.242 |
| ΔR^2^ | 0.014 | 0.346 | 0.040 | 0.126 | 0.038 | 0.119 | 0.001 | 0.798 | 0.022 | 0.242 |

*Note.* Adjusted for maternal age at baseline, pre-pregnancy BMI, gestation week at sampling, sleep time, and infant sex. AUC_G_ = area under the curve with respect to ground; AUC_I_ = area under the curve with respect to increase; β = standard coefficients; CI = confidence interval; T2 = second trimester.

**Supplementary Table 3.** *Cont.*

| **Melatonin rhythm during T3 (n = 70)** | | | | | | | | | | |
| --- | --- | --- | --- | --- | --- | --- | --- | --- | --- | --- |
| Variable | **Mean melatonin** | | **Amplitude** | | **Maximal level** | | **AUC_G_** | | **AUC_I_** | |
|  | β (95% CI) | *p*-value | β (95% CI) | *p*-value | β (95% CI) | *p*-value | β (95% CI) | *p*-value | β (95% CI) | *p*-value |
| **Meal frequency** | -0.20 (-6.39, 1.33) | 0.193 | -0.26 (-12.83, 2.67) | 0.191 | -0.14 (-13.39, 5.22) | 0.380 | -0.30 (-17.76, 0.28) | 0.057 | 0.06 (-10.06, 14.97) | 0.694 |
| ΔR^2^ | 0.034 | 0.193 | 0.055 | 0.191 | 0.017 | 0.380 | 0.078 | 0.057 | 0.003 | 0.694 |
| **Eating window** | **-0.40 (-4.07, 0.72)** | **0.006**** | -0.08 (-0.57, 0.33) | 0.581 | **-0.42 (-9.76, 1.72)** | **0.006**** | **-0.44 (-9.61, -2.09)** | **0.003**** | 0.10 (-4.75, 8.30) | 0.585 |
| ΔR^2^ | **0.144** | **0.006**** | 0.006 | 0.581 | **0.152** | **0.006**** | **0.173** | **0.003**** | 0.007 | 0.585 |
| **Breakfast-skipping** |  |  |  |  |  |  |  |  |  |  |
| No | 1.00 |  | 1.00 |  | 1.00 |  | 1.00 |  | 1.00 |  |
| Yes | 0.14 (-4.49, 11.00) | 0.400 | 0.11 (-12.77, 22.16) | 0.587 | 0.13 (-11.99, 25.90) | 0.462 | 0.15 (-10.70, 26.36) | 0.398 | -0.02 (-26.03, 22.46) | 0.882 |
| ΔR^2^ | 0.014 | 0.400 | 0.010 | 0.587 | 0.012 | 0.462 | 0.016 | 0.398 | 0.000 | 0.882 |
| **Late-night eating** |  |  |  |  |  |  |  |  |  |  |
| No | 1.00 |  | 1.00 |  | 1.00 |  | 1.00 |  | 1.00 |  |
| Yes | -0.24 (-11.31, 1.24) | 0.113 | 0.03 (-1.35, 1.65) | 0.841 | -0.26 (-27.75, 2.06) | 0.089 | -0.21 (-25.36, 4.56) | 0.168 | -0.02 (-20.79, 18.49) | 0.906 |
| ΔR^2^ | 0.050 | 0.113 | 0.001 | 0.841 | 0.061 | 0.089 | 0.042 | 0.168 | 0.000 | 0.906 |

*Note.* Adjusted for maternal age at baseline, pre-pregnancy BMI, gestation week at sampling, sleep time, and infant sex. AUC_G_ = area under the curve with respect to ground; AUC_I_ = area under the curve with respect to increase; CI = confidence interval; T3 = third trimester.

*p<0.05, **p<0.01.

**Supplementary Table 4.** Hierarchical linear regression predicting cortisol rhythm from chrononutrition characteristics during pregnancy.

| **Cortisol rhythm during T2 (n=70)** | | | | | | | | | | | |
| --- | --- | --- | --- | --- | --- | --- | --- | --- | --- | --- | --- |
| Variable | **Awakening cortisol** | | | **Mean cortisol** | | | **Amplitude** | | **Diurnal slope** | | |
|  | β (95% CI) | *p*-value | | β (95% CI) | | *p*-value | β (95% CI) | *p*-value | β (95% CI) | | *p*-value |
| **Meal frequency** | 0.15 (-3.43, 12.45) | 0.259 | | 0.12 (-1.37, 3.76) | | 0.354 | -0.20 (-1.25, 0.18) | 0.139 | 0.18 (-0.11, 0.80) | | 0.136 |
| ΔR^2^ | 0.021 | 0.259 | | 0.013 | | 0.354 | 0.036 | 0.139 | 0.032 | | 0.136 |
| **Eating window** | -0.02 (-4.88, 4.28) | 0.897 | | 0.06 (-1.11, 1.85) | | 0.619 | -0.12 (-0.59, 0.24) | 0.406 | 0.17 (-0.09, 0.43) | | 0.189 |
| ΔR^2^ | 0.000 | 0.897 | | 0.004 | | 0.619 | 0.011 | 0.406 | 0.025 | | 0.189 |
| **Breakfast-skipping** |  |  | |  | |  |  |  |  | |  |
| No | 1.00 |  | | 1.00 | |  | 1.00 |  | 1.00 | |  |
| Yes | **-0.33 (-25.37, -1.47)** | **0.029*** | | -0.07 (-6.34, 4.00) | | 0.653 | **0.43 (0.78, 3.55)** | **0.003**** | 0.19 (-0.34, 1.59) | | 0.198 |
| ΔR^2^ | **0.080** | **0.029*** | | 0.003 | | 0.653 | **0.135** | **0.003**** | 0.024 | | 0.198 |
| **Late night eating** |  |  | |  | |  |  |  |  | |  |
| No | 1.00 |  | | 1.00 | |  | 1.00 |  | 1.00 | |  |
| Yes | -0.06 (-15.37, 9.73) | 0.654 | | 0.14 (-1.75, 6.02) | | 0.275 | -0.04 (-1.37, 0.99) | 0.747 | -0.01 (-0.76, 0.71) | | 0.940 |
| ΔR^2^ | 0.003 | 0.654 | | 0.018 | | 0.275 | 0.002 | 0.747 | 0.000 | | 0.940 |
| Variable | **Morning slope** | | | | **Evening slope** | | | **AUC_G_** | | | |
|  | β (95% CI) | | *p*-value | | β (95% CI) | | *p*-value | β (95% CI) | | *p*-value | |
| **Meal frequency** | -0.01 (-0.97, 0.90) | | 0.942 | | 0.15 (-0.22, 0.76) | | 0.272 | 0.18 (-2.35, 11.72) | | 0.187 | |
| ΔR^2^ | 0.000 | | 0.942 | | 0.020 | | 0.272 | 0.027 | | 0.187 | |
| **Eating window** | 0.11 (-0.31, 0.75) | | 0.404 | | 0.00 (-0.29, 0.29) | | 0.998 | 0.02 (-3.83, 4.48) | | 0.874 | |
| ΔR^2^ | 0.010 | | 0.404 | | 0.000 | | 0.998 | 0.000 | | 0.874 | |
| **Breakfast-skipping** |  | |  | |  | |  |  | |  | |
| No | 1.00 | |  | | 1.00 | |  | 1.00 | |  | |
| Yes | -0.05 (-2.31, 1.66) | | 0.743 | | -0.19 (-1.69, 0.41) | | 0.225 | -0.22 (-24.88, 3.95) | | 0.151 | |
| ΔR^2^ | 0.002 | | 0.743 | | 0.025 | | 0.225 | 0.032 | | 0.151 | |
| **Late-night eating** |  | |  | |  | |  |  | |  | |
| No | 1.00 | |  | | 1.00 | |  | 1.00 | |  | |
| Yes | -0.06 (-1.87, 1.12) | | 0.617 | | 0.14 (-0.37, 1.21) | | 0.296 | 0.07 (-7.89, 13.82) | | 0.585 | |
| ΔR^2^ | 0.004 | | 0.617 | | 0.018 | | 0.296 | 0.005 | | 0.585 | |

*Note.* Adjusted for maternal age at baseline, pre-pregnancy BMI, household income level, gestation week at sampling, wake time, and infant sex. AUC_G_ = area under the curve with respect to ground; CI = confidence interval; T2 = second trimester. *p<0.05, **p<0.01.

**Supplementary Table 4.** *Cont.*

| **Cortisol rhythm during T3 (n=70)** | | | | | | | | | | | |
| --- | --- | --- | --- | --- | --- | --- | --- | --- | --- | --- | --- |
| Variable | **Awakening cortisol** | | | **Mean cortisol** | | | **Amplitude** | | **Diurnal slope** | | |
|  | β (95% CI) | *p*-value | | β (95% CI) | | *p*-value | β (95% CI) | *p*-value | β (95% CI) | | *p*-value |
| **Meal frequency** | 0.04 (-7.50, 9.57) | 0.809 | | -0.08 (-5.33, 3.01) | | 0.578 | -0.19 (-1.53, 0.39) | 0.235 | -0.06 (-0.99, 0.63) | | 0.655 |
| ΔR^2^ | 0.001 | 0.809 | | 0.006 | | 0.578 | 0.029 | 0.235 | 0.003 | | 0.655 |
| **Eating window** | 0.24 (-0.61, 7.39) | 0.094 | | 0.01 (-2.03, 2.14) | | 0.957 | -0.08 (-0.60, 0.35) | 0.593 | -0.22 (-0.66, 0.07) | | 0.116 |
| ΔR^2^ | 0.049 | 0.094 | | 0.000 | | 0.957 | 0.006 | 0.593 | 0.039 | | 0.116 |
| **Breakfast-skipping** |  |  | |  | |  |  |  |  | |  |
| No | 1.00 |  | | 1.00 | |  | 1.00 |  | 1.00 | |  |
| Yes | **-0.29 (-30.77, -0.41)** | **0.044*** | | -0.13 (-10.74, 4.39) | | 0.403 | -0.16 (-2.69, 0.88) | 0.312 | 0.27 (-0.07, 2.68) | | 0.062 |
| ΔR^2^ | **0.147** | **0.044*** | | 0.014 | | 0.403 | 0.021 | 0.312 | 0.055 | | 0.062 |
| **Late-night eating** |  |  | |  | |  |  |  |  | |  |
| No | 1.00 |  | | 1.00 | |  | 1.00 |  | 1.00 | |  |
| Yes | -0.09 (-18.12, 9.63) | 0.541 | | 0.10 (-4.56, 8.79) | | 0.516 | 0.04 (-1.35, 1.78) | 0.782 | 0.04 (-1.04, 1.40) | | 0.769 |
| ΔR^2^ | 0.007 | 0.541 | | 0.008 | | 0.526 | 0.002 | 0.782 | 0.001 | | 0.769 |
| Variable | **Morning slope** | | | | **Evening slope** | | | **AUC_G_** | | | |
|  | β (95% CI) | | *p*-value | | β (95% CI) | | *p*-value | β (95% CI) | | *p*-value | |
| **Meal frequency** | -0.31 (-5.46, 0.03) | | 0.052 | | -0.11 (-1.08, 0.48) | | 0.444 | 0.03 (-12.56, 14.92) | | 0.864 | |
| ΔR^2^ | 0.087 | | 0.052 | | 0.011 | | 0.444 | 0.001 | | 0.864 | |
| **Eating window** | 0.18 (-0.81, 2.28) | | 0.337 | | -0.09 (-0.47, 0.25) | | 0.535 | -0.02 (-6.94, 6.18) | | 0.908 | |
| ΔR^2^ | 0.023 | | 0.337 | | 0.007 | | 0.535 | 0.000 | | 0.908 | |
| **Breakfast-skipping** |  | |  | |  | |  |  | |  | |
| No | 1.00 | |  | | 1.00 | |  | 1.00 | |  | |
| Yes | 0.15 (-2.96, 7.20) | | 0.402 | | 0.21 (-0.42, 2.23) | | 0.177 | -0.27 (-44.80, 1.73) | | 0.069 | |
| ΔR^2^ | 0.017 | | 0.402 | | 0.034 | | 0.177 | 0.058 | | 0.069 | |
| **Late-night eating** |  | |  | |  | |  |  | |  | |
| No | 1.00 | |  | | 1.00 | |  | 1.00 | |  | |
| Yes | 0.05 (-4.19, 5.74) | | 0.753 | | -0.21 (-2.05, 0.33) | | 0.153 | -0.04 (-24.32, 17.90) | | 0.761 | |
| ΔR^2^ | 0.002 | | 0.753 | | 0.038 | | 0.153 | 0.002 | | 0.761 | |

*Note.* Adjusted for maternal age at baseline, pre-pregnancy BMI, household income level, gestation week at sampling, wake time, and infant sex. AUC_G_ = area under the curve with respect to ground; CI = confidence interval; T3 = third trimester. *p<0.05.
